# Left atrial myxoma with left ventricular myxoma diagnosed by ultrasound examination: A case report

**DOI:** 10.1097/MD.0000000000026903

**Published:** 2021-08-13

**Authors:** Xiang Ji, Xia Zhang

**Affiliations:** a Department of Ultrasound, Yancheng Dafeng People's Hospital, Yancheng, Jiangsu, P.R. China; b Department of Ultrasound, The First Affiliated Hospital of Wannan Medical College, Wuhu, Anhui, P.R. China.

**Keywords:** cardiac tumor, echocardiography, left ventricular myxoma, transthoracic echocardiography

## Abstract

**Rationale:**

Left ventricular (LV) myxoma is a rare type of benign cardiac tumor, which may result in unfavorable consequences due to embolism, arrhythmia, obstruction to the outflow tract, and other constitutional symptoms. LV myxoma can be easily misdiagnosed as LV thrombosis. Although some literatures have reported LV myxoma, the echocardiographic features of Left atrial (LA) myxoma with LV myxoma have rarely been reported till date. Here, we report case of LA myxoma with LV myxoma diagnosed by echocardiographic examination.

**Patient concerns:**

A 56-year-old male patient suffering from chest tightness and asthma for 6 months and progressive aggravation for 1 month was admitted to our hospital.

**Diagnosis:**

Echocardiographic imaging gave the suspicion of LA myxoma with LV myxoma, which was confirmed by pathology.

**Interventions:**

This patient was treated surgically.

**Outcomes:**

The patient had no postoperative complications and is currently under regular follow-up.

**Lessons:**

Echocardiography can be an effective imaging method for the evaluation of LV myxoma. The combination of echocardiography and clinical symptoms may help to make an accurate diagnosis.

## 1 Introduction

Cardiac myxoma is the most common primary cardiac tumor. Most of the myxomas are in the left atrium, but rarely seen in the left ventricle.^[1,2,3]^ Left atrial (LA) myxoma usually originates in atrial septum. However, Left ventricular (LV) myxoma could be located in any part of the left ventricle.^[4]^ LV myxoma is usually benign, but may lead to fatal consequences including emboli and cardiac arrhythmias resulting in sudden death.^[5,6]^ Because of the deadly outcome of this disease, it is important to make a correct diagnosis. LV myxoma, which is easily misdiagnosed as LV thrombosis, has been reported in some case reports. LA myxoma with LV myxoma is exceedingly rare. The echocardiographic features of LA myxoma with LV myxoma have rarely been reported till date. Here, we report case of LA myxoma with LV myxoma diagnosed by echocardiographic examination, pathological examination and other auxillary examination methods.

## 2 Case report

A 56-year-old male patient was admitted to our hospital on July 20, 2020. He had been diagnosed as LA myxoma in another hospital by echocardiography on July 17, 2020. He had a history of chest tightness and asthma for 6 months and progressive aggravation for 1 month, which disrupted the sleep. He had no headache, fever, palpitation, edema of lower limbs, and relevant family history. On physical examination, increased breath sounds were noted. Auscultation revealed a grade 3/ 6 tricuspid systolic murmur.

**Figure 1:**
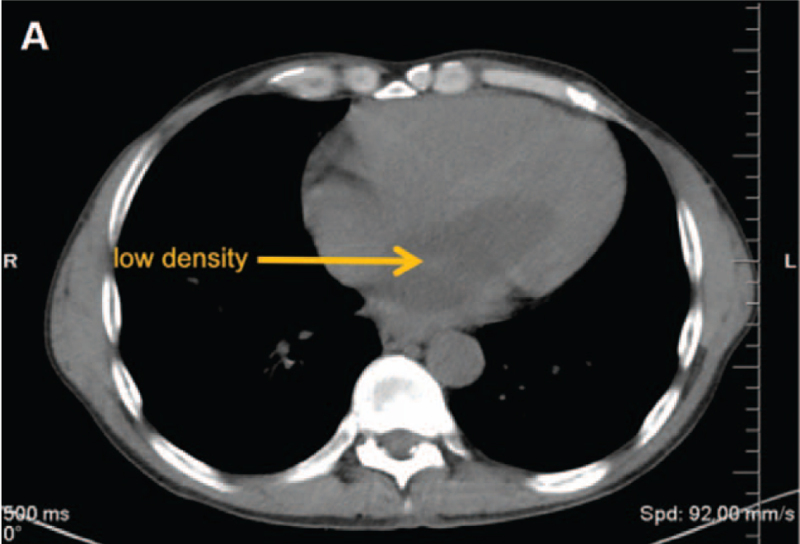
HRCT image of heart in this patient. (A) HRCT demonstrated a lesion with low density (yellow arrow) in the left heart. Cardiac enlargement was observed. HRCT = High-resolution computed tomography.

**Figure 2:**
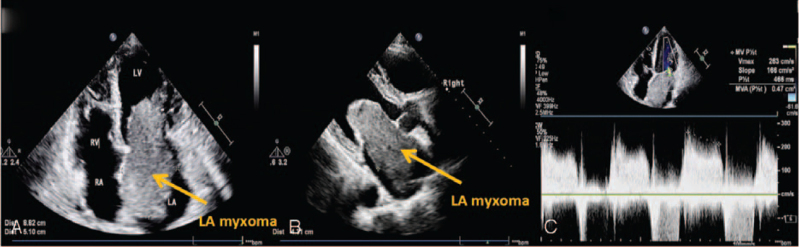
Echocardiographic imaging of LA myxoma in this patient. (A) Two-dimensional echocardiography revealed a mass of 88 mm in length and 51 mm in width (yellow arrow) in the left heart in the apical four chamber view. (B) Two-dimensional echocardiography showed a thick mass of 43 mm (yellow arrow) in the left heart in the apical two chamber view. (C) Pulse doppler imaging showed the accelerated forward flow of mitral valve. Mitral stenosis was noted. LA = Left atrial.

**Figure 3:**
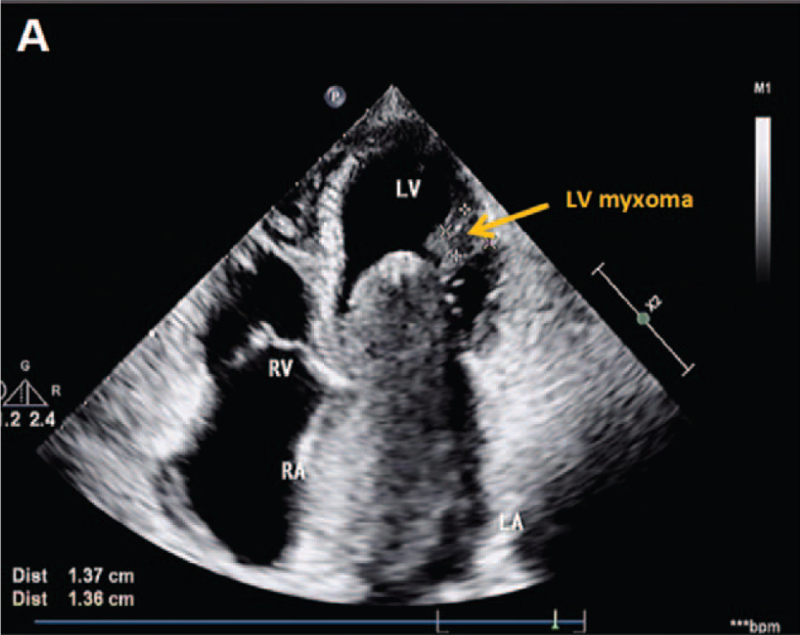
Echocardiographic imaging of LV myxoma in this patient. (A) Two-dimensional echocardiography revealed a mass of 14 mm in length and 14 mm in width (yellow arrow) in the left ventricle in the apical four chamber view. LV = Left ventricular.

On July 21, 2020, high-resolution computed tomography (HRCT) revealed that the cardiac enlargement and showed a lesion with low density in the left side of the heart. (Fig. 1). Transthoracic echocardiography (TTE) showed a medium echoic mass measuring 88 mm × 51mm × 43 mm in the left atrium. The mass was attached by a pedicle to the atrial septum and protruded into the left ventricle through the mitral orifice in diastole and returned to the atrium in systole, leading to obstruction of the mitral orifice during diastole. The cardiac atrium was enlarged, and the left ventricular ejection fraction (LVEF) was 65%. (Fig. 2). A medium echoic mass measuring 14 mm × 14 mm attached to the LV papillary muscle was also demonstrated by TTE. The mass moved back and forth in the left ventricle. (Fig. 3). This was suggestive of LV myxoma with LA myxoma. Additionally, severe tricuspid regurgitation was also found.

On July 22, 2020, surgery was performed on the patient which included tumor resection and tricuspid valvuloplasty. During the surgery, pericardium and right atrium were incised longitudinally. Next, part of the atrial septum was opened below the foramen ovale, and the atrial septum was pulled upward for exploration showing two tumors in the left heart, one of which was attached to the atrial septum, and the other was attached to the lateral wall of left ventricle. The two tumors looked crispy, gelatinous with complete capsule. The tumors and the tissues around their pedicles were resected completely. The tumor attached to the atrial septum measured approximately 80 mm × 53 mm × 43 mm, and the other attached to the lateral wall of left ventricle was about 14 mm × 14 mm × 12 mm. The tricuspid valve was then explored, and severe tricuspid regurgitation was found. Ten sutures were placed the valve ring, tricuspid annuloplasty ring was implanted, and no obvious regurgitation was found at this time. After the surgery, the patient was transferred to intensive care unit for close observation.

**Figure 4:**
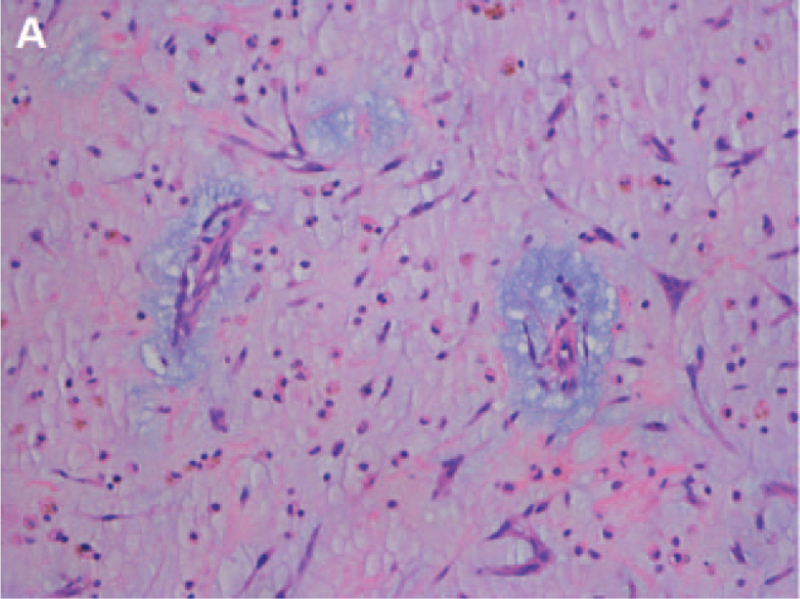
Microscopic appearance of myxoma. (A) Photomicrograph showed irregular shaped cells loosely dispersed within a mucoid ground substance. HE = hematoxylin-eosin staining 200 ×.

On a subsequent histopathological examination, photomicrograph showed irregular shaped cells loosely dispersed within a mucoid ground substance (Fig. 4). The postoperative diagnosis was LV myxoma with LA myxoma.

Tracheal intubation and ventilator-assisted breathing were given after the surgery. Ambroxol was used to prevent pulmonary complications, such as atelectasis; omeprazole was used to prevent stress ulcer, and reduced glutathione was used to protect liver function. Antibiotics were also administered. On postoperative day 2, extubation was performed when the patient was fully awake. On postoperative day 3, antibiotics were stopped, and cardiotonics and diuretics were given.

**Figure 5:**
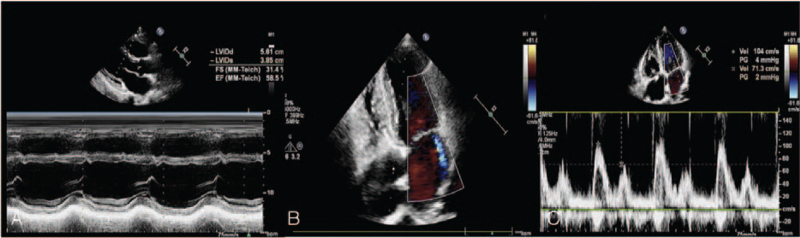
Echocardiographic imaging of this patient after operation. (A) M mode and two-dimensional echocardiography showed cardiac enlargement with no mass, and the LVEF was 59%. (B) Color doppler imaging showed mild mitral regurgitation; (C) Pulsed Doppler showed the normal forward flow velocity of mitral valve.

**Figure 6:**
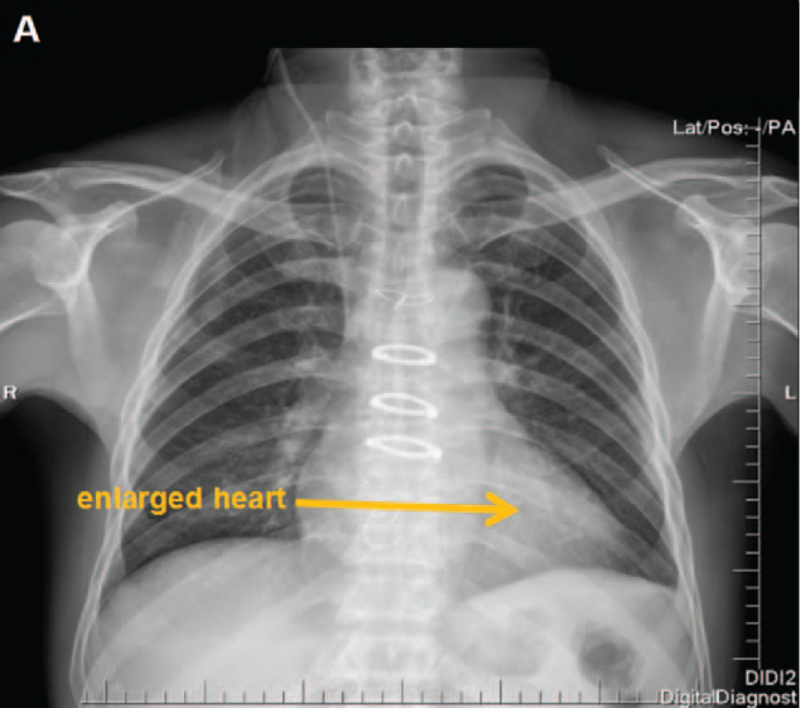
DR image of this patient after operation. (A) DR revealed cardiac enlargement (yellow arrow).

On postoperative day 6, TTE showed mild atrial enlargement, with a left ventricular ejection fraction (LVEF) of 59% and a mild regurgitation in mitral valve, which meant the patient's cardiac condition was well. (Fig. 5). The patient was told to rest adequately, avoid strenuous exercise and follow-up every six months. Digital Radiography (DR) revealed mild cardiac enlargement (Fig. 6).

On postoperative day 8, the patient 's incision healed well and there was no abnormality on physical examination. The patient was discharged on postoperative day 8, and no medication was required post-discharge. A one-year follow-up revealed no recurrence of the lesion, and we are still closely following the patient. The study protocol was approved by the ethics committee and institutional review board of the First Affiliated Hospital of Wannan Medical College [(2019) Ethics research No. 87]. Written informed consent was obtained from the patient for publication of the case details and accompanying images.

## 3 Discussion and conclusion

Cardiac myxoma is the most common primary cardiac tumor. Most of the cardiac myxomas are located in left atrium.^[7]^ The incidence rate of cardiac myxoma in female is higher than that in male. LV myxomas are very rare.^[8]^ Unlike LA myxomas, LV myxomas can be attached to any part of the left ventricle. In the literature review by Abad C et al,^[9]^ 24 of the 71 LV myxomas were attached to the ventricular septum and 46 of 71 originated in the left ventricular wall. Because of their mobile nature, LV myxomas may result in fatal consequences due to embolism, arrhythmia, obstruction to the outflow tract, and other constitutional symptoms.^[10,11]^ Surgical treatment should be performed once the diagnosis is confirmed.

Pathology is the gold standard for the diagnosis of myxoma. Cardiac myxoma typically present as a single, pedunculated, fragile, and irregular shaped lesion with an intact capsule. Histologically, cardiac myxomas are characterized by irregular or star shaped cells loosely dispersed within a mucoid ground substance and usually radially distributed around the small blood vessels.

Echocardiography is the first choice for early detection of myxoma and it is noninvasive, safe, and accurate. Although LV myxoma is rare, it is not difficult to make an accurate diagnosis according to its typical echocardiographic features. The echocardiographic features of LV myxoma present as a hyperechoic mass localized in the LV chamber. The mass moves back and forth in the left ventricle with the contraction and relaxation of the heart and may protrude into the left ventricular outflow tract (LVOT), leading to the obstruction to the outflow tract. LV myxoma should be differentiated from LV thrombus in echocardiographic characteristics. Compared with LV thrombosis, the typical LV myxoma has higher mobility and narrower base. Kong Y et al^[4]^ reported a TTE finding of LV myxoma leading to stroke. Through TTE, Qin W et al^[12]^ observed a LV myxoma attached at the base of the anterolateral papillary muscle in a patient with no history of syncope, palpitation, or dyspnea. Under the guidance of transesophageal echocardiography, Hamasaki A et al^[13]^ successfully resected the myxoma originating from the left ventricular wall. Transesophageal echocardiography (TEE) can provide more accuracy in the diagnosis of LV myxoma than TTE especially for the detailed information such as the morphology of the mass. The TEE features of left ventricular myxoma present as a pedunculated, homogeneous, and mobile mass, which could stem from any part of the left ventricle.^[13,14,15]^ During systole, the mass could protrude into the LVOT, leading to the obstruction of LVOT. Mitral valve prolapse and mild mitral regurgitation may be caused by the mass attached to the anterior lobe of the mitral valve.^[16]^ The TEE features of left atrial myxoma present as a mass attached to the atrial septum. The tumor could protrude into the left ventricle through the mitral valve and block the mitral valve during diastole.^[17,18]^ During systole, the mass returns to the left atrium. The left atrium may be enlarged due to the obstruction of the mitral valve by myxoma. Left ventricular thrombus is often located in the left ventricular apex. The TEE features of Left ventricular thrombus present as a mass with uneven echo, wide base and no pedicle. The mass is seen attached to the ventricular wall with its free surface pointed to the cardiac cavity. Left atrial thrombus is often located in the left atrial appendage. The TEE features of Left atrial thrombus present as a mass with low echo, wide base, and oval or irregular shape. There is no change in the shape of the mass during cardiac contraction and relaxation. In mitral stenosis and atrial fibrillation, thrombus may appear in any part of the left atrium. Baek SH et al^[16]^ misidentified the LV myxoma as an accessory mitral valve tissue by TTE, but on TEE the mass was accurately diagnosed as LV myxoma. Francois J et al^[14]^ showed a LV myxoma attached to chordeae tendinea in an asymptomatic patient through TEE.

The echocardiographic features of LA myxoma with LV myxoma have rarely been reported till date. In this case, the left atrial myxoma caused mitral valve obstruction, and presented as chest tightness. A LA myxoma attached to the atrial septum was detected through TTE with no difficulty. We also detected an irregular mass attached to the lateral wall of left ventricle that was considered LV thrombosis but later diagnosed as LV myxoma. A lack of history of organic heart disease or hypercoagulable state in the patient as well as the high mobility of the mass supported the diagnosis of LV myxoma.

Complications of the surgery of cardiac myxoma include left ventricular dysfunction, atelectasis, respiratory distress, infection, among others. Cardiac myxoma has a good postoperative prognosis, low recurrence rate and high long-term survival rate.^[19]^ Regular postoperative echocardiographic examination is required to evaluate for the recurrence of myxoma.

In conclusion, cardiac myxoma is the most common primary tumor of heart which may lead to embolic events. LV myxoma is a type of extremely rare cardiac tumor which is easily misdiagnosed as thrombosis. LA myxoma could be accompanied by LV myxoma. Echocardiography is an important tool in the diagnosis of cardiac myxoma. The combination of echocardiography and clinical manifestations may help to make the accurate diagnosis.

## Acknowledgments

We thank the patient and his family for their participation in this study.

## Author contributions

**Conceptualization:** Xia Zhang.

**Data curation:** Xiang Ji.

**Funding acquisition:** Xia Zhang.

**Investigation:** Xiang Ji.

**Software:** Xiang Ji.

**Writing – original draft:** Xiang Ji.
